# Genital *Chlamydia trachomatis* Infections Clear More Slowly in Men Than Women, but Are Less Likely to Become Established

**DOI:** 10.1093/infdis/jix283

**Published:** 2017-06-13

**Authors:** Joanna Lewis, Malcolm J Price, Paddy J Horner, Peter J White

**Affiliations:** 1 National Institute for Health Research (NIHR) Health Protection Research Unit in Modelling Methodology and Medical Research Council Centre for Outbreak Analysis and Modelling, Imperial College London School of Public Health;; 2 Modelling and Economics Unit, National Infection Service, Public Health England, London;; 3 Institute of Applied Health Research, University of Birmingham; and; 4 NIHR Health Protection Research Unit in Evaluation of Interventions, University of Bristol, United Kingdom

**Keywords:** chlamydia, natural history, sexually transmitted diseases, Bayesian inference, evidence synthesis

## Abstract

**Background:**

Rigorous estimates for clearance rates of untreated chlamydia infections are important for understanding chlamydia epidemiology and designing control interventions, but were previously only available for women.

**Methods:**

We used data from published studies of chlamydia-infected men who were retested at a later date without having received treatment. Our analysis allowed new infections to take one of 1, 2, or 3 courses, each clearing at a different rate. We determined which of these 3 models had the most empirical support.

**Results:**

The best-fitting model had 2 courses of infection in men, as was previously found for women: “slow-clearing” and “fast-clearing.” Only 68% (57%–78%) (posterior median and 95% credible interval [CrI]) of incident infections in men were slow-clearing, vs 77% (69%–84%) in women. The slow clearance rate in men (based on 6 months’ follow-up) was 0.35 (.05–1.15) year^-1^ (posterior median and 95% CrI), corresponding to mean infection duration 2.84 (.87–18.79) years. This compares to 1.35 (1.13–1.63) years in women.

**Conclusions:**

Our estimated clearance rate is slower than previously assumed. Fewer infections become established in men than women but once established, they clear more slowly. This study provides an improved description of chlamydia’s natural history to inform public health decision making. We describe how further data collection could reduce uncertainty in estimates.

## Summary

Rigorous estimates for untreated chlamydia clearance rates are important for understanding and controlling infection. We synthesized literature data to investigate clearance in men. Established infections clear more slowly in men than women, and more slowly than had previously been assumed.

Chlamydia is the most commonly diagnosed sexually transmitted infection in many countries and can have serious sequelae, especially in women, including pelvic inflammatory disease leading to ectopic pregnancy and infertility [1]. Widespread testing has been recommended in countries including Australia, England, the Netherlands, Sweden, and the United States. However, there is uncertainty about the effectiveness of testing programs as control measures [2]. Although randomized controlled trials have provided evidence that screening can reduce the incidence of pelvic inflammatory disease [3, 4], a recent trial of chlamydia screening in the Netherlands observed no reduction in chlamydia prevalence [5, 6]. In young people in England, prevalence was similar in 1999–2001 and 2010–2012 [7], despite the rollout of the National Chlamydia Screening Programme (NCSP) from 2003, with full implementation by 2008. A better understanding of all aspects of chlamydia’s natural history and epidemiology is required in order to plan and implement reliably effective control measures that interrupt transmission by finding and curing infections that would otherwise be untreated.

Mathematical modeling studies are increasingly common tools for understanding the epidemiology of sexually transmitted infections and planning and evaluating public health interventions [8–14]. Results and conclusions from these models are highly sensitive to their underlying assumptions, including the numerical values used to describe infection natural history [15, 16]. In models of chlamydia transmission and control, an important parameter is the clearance rate of untreated infections [15]. The more slowly clearance occurs, the higher the prevalence of untreated infection, and the more effective a screening intervention is likely to be. Conversely, if untreated infection is typically short-lived then intensive, active case-finding including partner notification will be required to shorten the duration sufficiently to have an impact on prevalence and transmission. The duration of untreated chlamydia is a difficult quantity to measure, as the standard of care is to treat individuals in whom infection has been detected. Perhaps for this reason, a wide range of durations have been assumed in modeling studies, ranging from 180 days to 2–3 years in men and women [17]. Many estimates currently used in mathematical models are based on short-term studies, cohorts where participants were treated at the time of the first test for a concurrent infection, or even the natural history of other infections [17].

Some of the most informative data on the duration of untreated infection comes from studies in which diagnosed cases were retested at a later date, having received no treatment between the 2 tests. If the time between testing and retesting is known, then the proportion of cases who had recovered in the interval provides information on the recovery rate. A recent report showed how this type of evidence from multiple studies could be synthesized to estimate the rate of chlamydia clearance and mean duration of infection in women [18]. Here, we apply the same method to estimate the rate of chlamydia clearance in men.

## METHODS

We used a mixture-of-exponentials model [18] for the duration of untreated chlamydia infection in men. The model allows for any new infection to take one of several courses, each with a different clearance rate. Each infection thus belongs to one category, or “class.” Within each class, durations of infection are exponentially distributed with mean equal to the reciprocal of the clearance rate for that class. To determine the appropriate number of classes, we compared the fit to the data of models comprised of 1, 2, and 3 classes. We also investigated a model in which clearance rates had a continuous Gamma distribution, rather than falling into discrete classes. The model also recognizes different study types: clinic-based studies recruit individuals who present for testing, perhaps because symptoms have recently developed or because of contact with an infected person. They therefore represent recent exposures and incident infections. Screening studies recruit members of the general population, more representative of prevalent infections. The proportion of detected infections belonging to faster-clearing infection classes is lower in screening studies than in patients seeking care after exposure to infection because these short infections clear quickly and, thus, are less common in the pool of prevalent infections.

We used 3 published literature reviews to identify relevant studies in which chlamydia-infected men were retested at a later date, having received no treatment in the interim [19–21]. We updated these searches to cover the period since the original review but found no further relevant evidence. The search strategy for these updates is provided online at https://github.com/joanna-lewis/ct_clearance. The studies provide information on natural (rather than antibiotic-induced) chlamydia clearance. In some cases, this formed the placebo or no-treatment arm of a comparison between different drugs; in others, it represented the delay between collecting a sample and the patient returning for treatment. In 1 case, patients were diagnosed during an army medical examination, and treatment was not actively sought by, or offered to, the asymptomatic patients.

Eight studies were included and 165 men were retested altogether, at a total of 18 time points after the original diagnostic test. Six studies (13 time points) were clinic-based and 2 (5 time points) were screening studies. Five studies (11 time points) used culture diagnosis methods; 3 (7 time points) used nucleic acid amplification tests (NAATs). Six (12 time points) diagnosed infection using urethral swabs, and 2 (6 time points) used urine samples. The data are summarized in Table 1. Citations for the studies used are provided online with the search strategy.

**Table 1.** Data Sources for the Duration of Untreated Chlamydia Infection in Men

First Author, Publication Year^a^	Study Design; Sample Type; Diagnosis Method	Follow-up Period	Estimated Mean Follow-up, y	No. Tested at Follow-up	No. Clearing CT Infection	Crude Clearance Rate, Year^-1^, Mean (95% CI)
Handsfield, 1976	Clinic; Urethral; Culture	1 wk (0.019 y)	0.019	10	0	0 (0–19.4)
Prentice, 1976	Clinic; Urethral; Culture	7–21 d (0.019–0.057 y); mean, 8.5 d (0.023 y)	0.023	13	4	16.0 (4.1–41.4)
Johannisson, 1979	Clinic; Urethral; Culture	1 wk (0.019 y)	0.019	17	3	10.2 (2.0–30.0)
		2 wk (0.038 y)	0.038	27	13	17.3 (8.9–30.0)
		3 wk (0.058 y)	0.058	6	3	12.0 (2.2–36.8)
		4 wk (0.077 y)	0.077	2	2	NA (2.2–NA)
Paavonen, 1980	Clinic; Urethral; Culture	4 wk (0.077 y)	0.077	21	7	5.3 (2.0–11.0)
Joyner, 2002	Clinic; Urine; NAAT	2–7 d (0.005–0.019 y)	0.012	15	3	18.6 (3.7–54.6)
		8–14 d (0.022–0.038 y)	0.030	9	2	8.4 (1.0–30.6)
		15–21 d (0.041–0.057 y)	0.049	4	1	5.9 (.1–33.5)
		22–42 d (0.060–0.115 y)	0.088	4	0	0 (0–10.5)
		43–112 d (0.118–0.307 y)	0.190	4	1	1.5 (.0–8.6)
Geisler, 2008	Clinic; Urethral; NAAT	4–59 d (0.011–0.162 y)^b^	0.045	14	5	9.8 (3.0–23.2)
Stamm, 1986	Clinic-based screening; Urethral; Culture	1 wk (0.019 y)	0.019	5	1	11.7 (.3–66.3)
		2 wk (0.038 y)	0.038	2	0	0 (0–48.5)
		3 wk (0.058 y)	0.058	2	0	0 (0–31.8)
		4 wk (0.077 y)	0.077	1	0	0 (0–47.9)
van den Brule, 2002	Screening; Urine; NAAT	6 mo (0.5 y)	0.500	9	1	0.2 (.0–1.3)

Crude clearance rate is calculated using the formula –ln(1 – *θ*) / t, where *t* is the mean follow-up time and *θ* is the proportion of men having cleared infection. NA appears in the column for crude clearance rate where all of the men had cleared infection. The estimate and lower bound of the 95% confidence interval for *θ* were therefore zero, and the corresponding clearance rates were infinite. Intuitively this corresponds to the fact that if all men clear infection before observation, then there is the possibility that clearance is immediate. Mean follow-up for each time point was estimated as described by Price et al [18].

Abbreviations: CI, confidence interval; CT, *Chlamydia trachomatis*; NA, not available; NAAT, nucleic acid amplification test.

^a^See online information for full citation.

^b^Follow-up period for men and women combined.

The studies were of moderately good quality. Most were prospective follow-up of infected patients. While older studies were often randomized clinical trials with a placebo arm, in more recent studies the only patients who were not treated presumptively were those without indications or infection risk factors such as urethritis or an infected partner. Several studies had <100% follow-up, and patients with persistent symptoms may have been more likely to return, causing a bias toward slow clearance rates. Potential confounders, which would have opposing effects, are unreported treatment and reinfection in the interval between the first and second tests. However, both of these would be expected to be rare over the periods of observation; studies of chlamydia reinfection in heterosexual men after treatment have estimated rates of around 10% per year [22, 23]. These studies provide the best evidence available to inform estimates of the clearance rate of untreated infection.

The data inform parameter estimates for the mixture-of-exponentials model in a Bayesian inference framework. We found that the inference algorithm performed well only for models of duration consisting of 1 or 2 classes and not for 3 or more, so we did not investigate models of 3 or more classes any further. The clearance rate of short-duration (fast-clearing) infections proved difficult to identify, and was therefore fixed. Sensitivity analysis showed that estimates of the other parameters are insensitive to this value. To assess goodness-of-fit, we compared models with different numbers of infection classes, and different fixed values for the fast clearance rate, using the residual deviance and deviance information criterion (DIC) [24]. In a well-fitting model, the residual deviance is expected to approximate the number of data points. The DIC adjusts deviance to allow for model complexity, and a lower DIC indicates a better fit. We used uninformative prior distributions for the proportion of infections that are slow-clearing (Beta(1,1) distribution) and for the clearance rate of slow-clearing infection (Exp(0.001) distribution). As in the analysis for women [18], the prior distribution for the sensitivity of culture diagnosis methods was informed by analysis of samples from men and women who had previously tested positive by culture and were retested using culture and NAATs [25]. In a large study comparing the sensitivity of culture to NAAT diagnosis methods using different sample types [26], there was no evidence of a difference in the sensitivity of culture methods between urethral and cervical samples (113/146 urethral vs 102/125 cervical samples culture positive; χ^2^ ~ 10^–30^; *P* ≈ 1); nor was there a significant difference between urethral and male urine samples (113/146 urethral vs 112/145 urine samples culture positive; χ^2^ = 0.492; *P* = .483).

Posterior probability distributions for the 2 parameters (the proportion of infections clearing slowly, and the slow clearance rate) were obtained by Markov chain Monte Carlo sampling using the Stan system, via the rstan package [27] in the R statistical environment [28]. We also reproduced the analysis in women [18], to compare the posterior parameter distributions for men and women. We used the samples from the posterior distributions in each sex to derive samples for the difference in parameter values, by taking samples in turn and subtracting one from the other. Full details of the mathematical and statistical models are given in the R and Stan code used, which can be downloaded from https://github.com/joanna-lewis/ct_clearance.

## RESULTS

**Figure 1.**
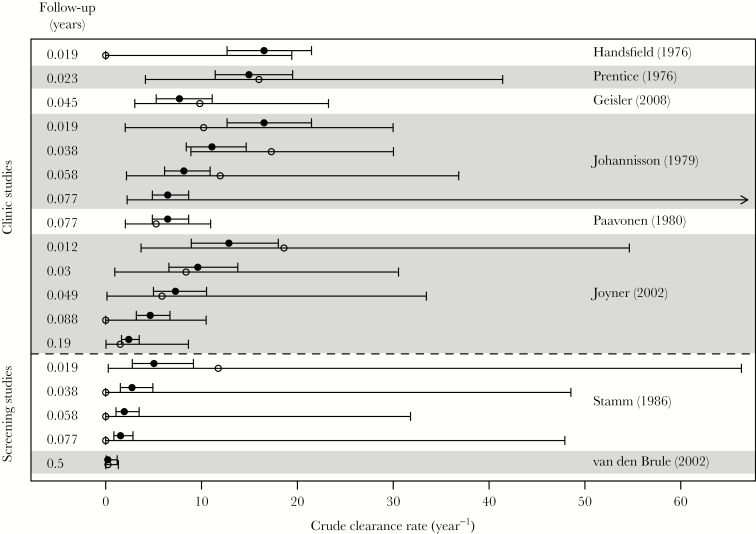
Validation of the model, showing crude (data; open circles) and simulated (model; filled circles) clearance rate for each data point in Table 1. Error bars give the 95% confidence interval for crude estimates, and the central 95% of simulations. For crude estimates, an arrow pointing right indicates that all men cleared the infection, so there is no upper bound or estimate for the crude clearance rate (see also the legend to Table 1). The first 6 studies (above the dashed line) were clinic-based, and the last 2 (below the line) were screening studies. Within this grouping, studies are ordered by maximum duration of follow-up. Follow-up in years at each observation is indicated on the left-hand side.

The best-fitting model has 2 classes of infection, fast- and slow-clearing. This 2-class model provided a better description of the data (DIC = 23.3) than a model with 1 infection class and 1 constant clearance rate (DIC = 59.0), or a model in which clearance rates had a Gamma distribution (DIC = 31.8). Figure 1 shows a validation of the model, in which the crude clearance rate estimated using each data point in Table 1 is plotted alongside predictions from the mixture-of-exponentials model. The good agreement between observations and predictions shows that our hypothesis of 2 clearance rates can explain the discrepancies between the crude clearance rates in Table 1.

The posterior mean for the proportion of infections in the slower-clearing category was 0.677, and the median was 0.677 (95% credible interval [CrI], .573–.779). For fast-clearing infections, we found that a clearance rate of 49 year^-1^, corresponding to mean duration 7.45 days, gave the best fit to data (ie, minimized the DIC). The posterior mean residual deviance was relatively high (21.6, compared with 18, the number of data points). For slow-clearing infections, the rate of chlamydia clearance was 0.35 (.05–1.15) year^-1^ (median, 95% CrI); these quantiles correspond to mean infection durations 2.84 (.87–18.7) years assuming that the slow clearance rate remains constant beyond the 6-month range of the data. The very wide upper bound on the mean duration (lower bound on clearance rate) is due to the scarcity of long-term data.

**Figure 2.**
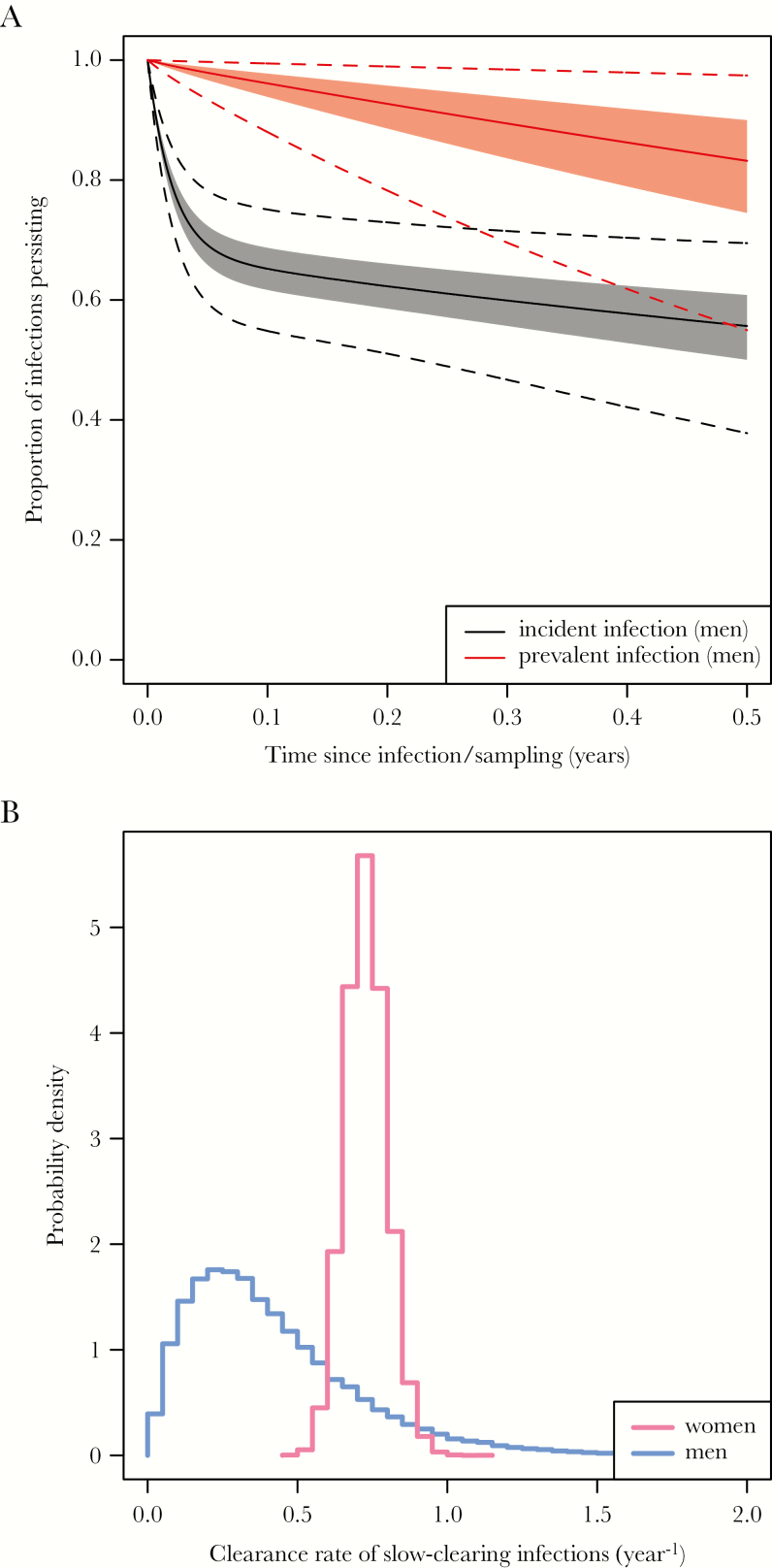
*A*, Simulated survival curves for incident and prevalent infections in men. Solid lines indicate the median of the simulated proportion persisting, shaded areas the central 50% (the interquartile range), and dashed lines the central 95%. Note that the time axis is time since infection for incident infections, and time since detection of infection for prevalent infections. *B*, Posterior distributions for the clearance rate of slow-clearing chlamydia infections in men (blue) and women (pink), based on follow-up of up to 6 months in men and 4 years in women.

Figure 2A uses the sampled parameter values to predict the survival of an incident infection (time zero equals the time of infection) and a prevalent infection (time zero equals the time of sampling from the population). Our posterior parameter distributions imply that prevalent infections are overwhelmingly slow-clearing, so that the duration of a prevalent infection is close to the equivalent for a slow-clearing infection. Figure 2B shows the sampled posterior distribution for the clearance rate of slow-clearing infections in men, with the corresponding distribution for women [18] for comparison. The distribution for men lies to the left of that for women, indicating that the clearance rate in men is likely to be slower than in women. The posterior for men is wider than that for women because of the shorter maximum follow-up, the smaller number of time points, and the smaller numbers of men than women tested at each time point.

**Figure 3.**
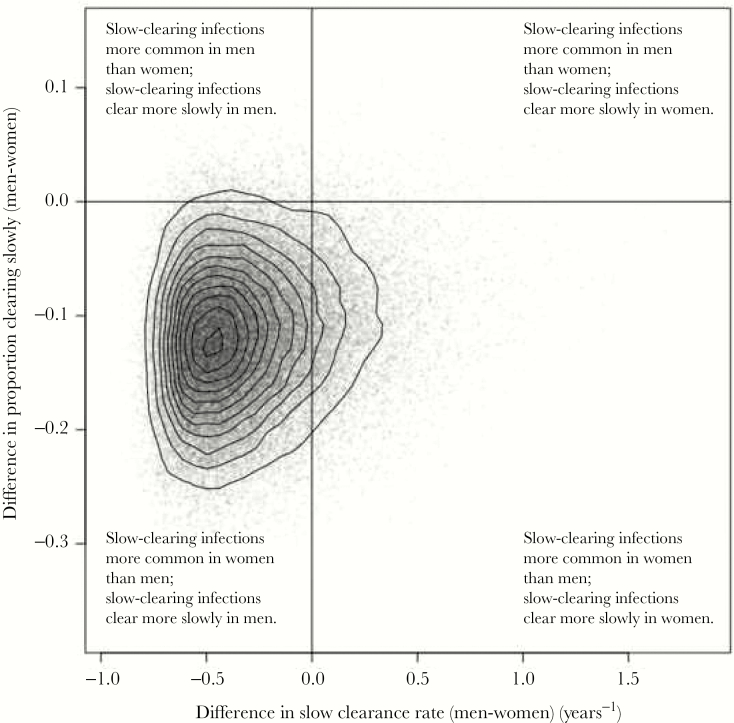
Posterior distribution for the difference between parameters characterizing infections in men and women. The x-axis represents differences in clearance rate and the y-axis represents differences in the proportion of infections that are slow-clearing. Solid lines at x = 0 and y = 0 divide samples in which parameters are higher/lower in men vs women.

Individual posterior samples for women were subtracted from individual samples for men to provide sampled distributions for the differences between the sexes in the proportion of incident infections clearing slowly, and the rate of slow clearance. These samples for the differences are plotted in Figure 3. The probability that episodes of slow-clearing infection clear more quickly in women than in men was 86% (the proportion of samples falling on the left-hand side of the plot). The probability that incident infections are more likely to be slow-clearing in women than in men was 98% (the proportion of samples in the lower part of the plot). There is strong evidence (84% of samples in the bottom-left quadrant) that infections are more likely to be slow-clearing in women and these slow-clearing infections persist longer in men.

## DISCUSSION

Our analysis is the first rigorous synthesis of the data available on the duration of untreated chlamydia infection in men. It indicates that incident chlamydia infections in men fall into fast- or slow-clearing classes, as has been reported for women [18]. It is often assumed that untreated infections clear at 1 constant rate [17], which we have now shown is an oversimplification for men as well as for women. Furthermore, we find that slow-clearing infections in men generally have a lower clearance rate than in women, the opposite of what has usually been assumed [17], but that exposure in men is less likely to result in an established infection. In countries reporting chlamydia testing, men test less frequently than women and fewer male infections are identified. Prolonged untreated infection in men means that identifying and treating infected men is an important priority for reducing transmission. We recommend greater focus on partner notification, to identify those persistently infected men who would be unlikely to receive treatment otherwise. Failure to treat men’s infections limits the effectiveness of testing in women because even when infected women are found and treated, they become reinfected from the undetected infection reservoir in men.

It should be noted that our use of the reciprocal of the slow clearance rate to estimate the mean duration of slow-clearing infections depends on the assumption that the clearance rate remains constant beyond the 6-month follow-up available in the data. In the absence of other information this is a reasonable, parsimonious assumption, but one that should be tested in future analyses of longer-term studies. Although the analysis took into account the imperfect sensitivity of culture diagnosis methods, it did not account for the possibility that an initially positive NAAT test could have detected DNA from dead bacteria, rather than viable organism. However, we would not expect this effect to affect the estimated slow clearance rate, and indeed sensitivity analysis (see online code at https://github.com/joanna-lewis/ct_clearance) shows that there is no substantial evidence of a difference in slow clearance rate between the culture-based and NAAT-based studies. We also repeated the analysis excluding each study in turn to investigate whether any study might be affecting the results unduly, but found that only the removal of the 6-month study had a marked impact on the posterior distribution for duration of infection. This impact was a result of the longer follow-up in this study, rather than any difference in the quality of the evidence.

Our estimated duration of untreated infection of 2.84 years in men suggests that the durations of up to 200 days that are commonly assumed in modeling studies [17] are much too short. Persistent infection in men is consistent with evidence from several other studies. The Chlamydia Screening Studies (ClaSS) found there was no reduction in chlamydial load in urine samples from infected men retested between 14 and 62 days after diagnosis (median, 23.5 days) [29]. Chlamydia has been detected in semen from sperm donors at multiple time points over the course of 2 years [30], although this could represent repeat infections. A couple study of 1690 asymptomatic women and their male sex partners in which the mean duration of sexual partnership ranged from 2 months to >10 years observed that chlamydia was detected more often in men than women [31], which is consistent with the duration of asymptomatic infection being longer in men than women. Finally, there is also population-level evidence that untreated infection lasts longer in men than women. Prior to the NCSP in England, prevalence in men and women was similar [32]. Infected women made fewer contacts than infected men [32] and the transmission probability from women to men is probably no higher than from men to women [17], suggesting that incidence was lower in men. Lower incidence could only result in a similar prevalence if the duration of infection is correspondingly longer.

The finding that there are fast- and slow-clearing infections in men as well as women, and the quantitative differences between men and women, are consistent with what is known about the biology of *C. trachomatis* and the human immune response. An explanation for the higher proportion of fast-clearing infections in men may be that urination makes it more difficult for the bacterium to remain present for long enough to penetrate the mucin barrier lining the urethra, allowing cell attachment and infection. This would not be the case with the female cervical epithelium, which continues to be exposed to chlamydia elementary bodies from the male ejaculate present in the vaginal fluid. The longer duration of established (slow-clearing) infections in men than in women may be explained by differences in sex hormones, which affect the immune response. Estradiol mainly enhances the immune response whereas testosterone is generally immunosuppressive [33]. This would be consistent with the observation that chlamydia antibodies are detected less often in men following infection than in women, although the precise role of antibody in resolving infection is unclear [34, 35] and human and animal studies suggest that the cellular immune response is also important for protection [36, 37].

Until the uncertainty in the clearance rate of chlamydia that we have quantified here has been reduced by further study, it is important that it should be recognized in statistical and modeling analyses [15, 16]. We need a better understanding of the duration of untreated chlamydia, and indeed of other sexually transmitted bacterial infections: this duration is also poorly characterized for gonorrhea [38] and *Mycoplasma genitalium* [39, 40], and reliable estimates are important for informing decisions about whether to test for and treat this latter emerging infection. As infected individuals should be treated promptly, natural history cannot be followed directly but valuable additional information could come from more studies retesting infected individuals, for example when they return to healthcare for treatment after testing positive. Large-scale public health programs provide an ideal opportunity to do this in a prevalent population, and the very fast clearance rates of short infections (estimated as 49 year^-1^ and 120 year^-1^ in men and women, respectively) mean that even as screening programs are extended, the majority of infections detected this way will still be prevalent rather than incident. England’s NCSP [41] monitors the delay between testing and treatment of infected patients, enabling patients with the longest delays, who would contribute most additional statistical information to our estimates, to be selected for retesting. It would be important to obtain information from these patients on the reason for the delay. For example, a patient who recovered and was then reinfected might have experienced the disappearance and then return of symptoms, prompting retesting. Linkage of patient records would allow studies examining whether the association between faster natural clearance of infection and lower rates of reinfection, reported by Geisler et al [42] for mostly African American women in a sexually transmitted disease clinic setting, occurs in other populations. Additionally, as the gastrointestinal tract may be an important reservoir for chlamydia infection in women because of the possibility of autoinfection [43, 44], rectal testing could be used to obtain estimates of the duration of rectal infection. A strength of the Bayesian approach that we have used is that different types of information can be incorporated as they become available to improve estimates in the future. For example, data from whole-genome sequence analysis could provide a lower-bound estimate for the duration of infection by informing on the interval between acquisition of infection and onward transmission, as has been recently applied to gonorrhea [45].

We have provided new, statistically rigorous estimates for the duration of untreated chlamydia infection in men, synthesizing data from several studies. The estimates add to a growing understanding of chlamydia epidemiology [46] and natural history [1, 42], which is essential if we are to make improved public health decisions. Given the importance of knowing the duration of untreated infection for understanding the natural history of chlamydia and other infections, we recommend further study in both men and women, including extragenital infections. We recommend that large-scale screening programs retest individuals returning for treatment after testing positive, particularly those patients with long delays, who are the most informative cases. This needs to be considered urgently, before implementation of point-of-care testing closes the gap between testing and treatment, removing this opportunity to deepen our understanding and improve public health.

## Notes

***Disclaimer.*** The funders had no role in study design, data collection and analysis, decision to publish, or preparation of the manuscript. The views expressed are those of the authors and not necessarily those of the Department of Health, Medical Research Council (MRC), National Health Service, National Institute for Health Research (NIHR), or Public Health England (PHE).

***Financial support.*** J. L. and P. J. W. were supported by the NIHR Health Protection Research Unit (HPRU) in Modelling Methodology at Imperial College London in partnership with PHE (grant number HPRU-2012–10080). P. J. W. was also supported by the Medical Research Council (MRC), through Centre funding (grant number MR/K010174/1). P. J. H. was supported by the NIHR HPRU in Evaluation of Interventions at the University of Bristol in partnership with PHE (grant number HPRU-2012–10026).

***Potential conflicts of interest.*** P. J. H. has received personal fees from the Crown Prosecution service and British Association for Sexual Health and HIV, travel funding from Hologic related to a study of *Trichomonas vaginalis*, and research funding from Mast Group Ltd for evaluation of a multiplex polymerase chain reaction assay. P. J. H. also reports a patent for a sialidase spot test to diagnose bacterial vaginosis. All other authors report no potential conflicts. All authors have submitted the ICMJE Form for Disclosure of Potential Conflicts of Interest. Conflicts that the editors consider relevant to the content of the manuscript have been disclosed.
